# Pharmacogenetic Biomarkers of Ibrutinib Response and Toxicity in Chronic Lymphocytic Leukemia: Insights from an Observational Study

**DOI:** 10.3390/ph18070996

**Published:** 2025-07-02

**Authors:** Noelia Pérez-Gómez, Antonio Sanz-Solas, Beatriz Cuevas, María Victoria Cuevas, Cristina Alonso-Madrigal, Javier Loscertales, Rodolfo Álvarez-Nuño, Covadonga García, Pablo Zubiaur, Gonzalo Villapalos-García, Raúl Miguel Parra-Garcés, Gina Mejía-Abril, Raquel Alcaraz, Raquel Vinuesa, Francisco Javier Díaz-Gálvez, María González-Oter, Natalia García-Sancha, Raúl Azibeiro-Melchor, Tomás José González-López, Francisco Abad-Santos, Jorge Labrador, Miriam Saiz-Rodríguez

**Affiliations:** 1 Research Unit, Fundación Burgos por la Investigación de la Salud (FBIS), Hospital Universitario de Burgos, 09006 Burgos, Spain; 2 Facultad de Ciencias de la Salud, Universidad de Burgos, 09001 Burgos, Spain; 3 Pharmacology Department, Facultad de Medicina, Universidad Autónoma de Madrid, 28049 Madrid, Spain; 4 Hematology Department, Hospital Universitario de Burgos, 09006 Burgos, Spain; 5 Hematology Department, Hospital Santiago Apóstol, Miranda de Ebro, 09200 Burgos, Spain; 6 Hematology Department, Hospital Universitario de La Princesa, 28006 Madrid, Spain; 7 Grupo 32, Instituto de Investigación Sanitaria La Princesa (IP), Hospital Universitario de La Princesa, 28006 Madrid, Spain; 8 Clinical Pharmacology Department, Hospital Universitario de La Princesa, Universidad Autónoma de Madrid, Instituto de Investigación Sanitaria La Princesa (IP), 28006 Madrid, Spain; 9 Cancer Genetics Group, Unit of Excellence Institute of Biomedicine and Molecular Genetics, University of Valladolid Spanish National Research Council (IBGM; UVa-CSIC), 47003 Valladolid, Spain

**Keywords:** chronic lymphocytic leukemia, ibrutinib, pharmacogenetics, polymorphisms

## Abstract

**Background/Objectives:** Ibrutinib is a selective Bruton’s tyrosine kinase inhibitor approved for the treatment of chronic lymphocytic leukemia (CLL). This drug exhibits significant variability in response and toxicity profile, possibly due to genetic polymorphisms in drug-metabolizing enzymes and transporters. The aim of this observational study is to address interindividual variability in the efficacy and safety of ibrutinib treatment in 49 CLL patients. **Methods:** Genotyping of nine polymorphisms was performed by quantitative polymerase chain reaction (qPCR) using a ViiA7^®^ PCR Instrument and TaqMan assays, and ibrutinib plasma concentrations were determined using high-performance liquid chromatography coupled to a tandem mass spectrometry detector (HPLC-MS/MS). **Results:** Our study confirmed a high response rate, with 62% of patients achieving complete remission (CR), 9% CR with incomplete hematologic recovery (CRi), and 24% partial remission (PR). The impact of genetic polymorphisms on the CR rate was evaluated, revealing no statistically significant associations for *CYP3A4*, *CYP3A5*, *ABCB1*, *ABCG2*, and *SLCO1B1* variants. However, a tendency was observed for patients carrying *ABCB1* rs1128503, rs1045642 T/T, or rs2032582 A/A genotypes to achieve a higher CR rate. Adverse drug reactions (ADRs) were frequent, with vascular disorders (39%) and infections (27%) being the most common. Genetic polymorphisms influenced ibrutinib toxicity, with *CYP3A4* *1/*22 appearing to be protective against overall ADRs. **Conclusions:** The unexpected association between *CYP3A4* *1/*22 genotype and lower ADR incidence, as well as the trend toward improved treatment response in patients carrying *ABCB1* genotypes, suggests compensatory metabolic mechanisms. However, given the small sample size, larger studies are needed to confirm these findings and their clinical implications, while also aiming to uncover other non-genetic factors that may contribute to a better understanding of the variability in treatment response and toxicity.

## 1. Introduction

B cell antigen receptor (BCR) signaling is one of the major pathways in the formation of lymphoid malignancies. Antigen stimulation of normal B cells induces BCR dimerization that triggers a downstream signaling cascade of kinases, which in turn regulates multiple cellular processes such as proliferation, differentiation, apoptosis, and survival [1].

Bruton’s tyrosine kinase (BTK) is an essential enzyme in the BCR signaling pathway and thus a promising therapeutic target for the treatment of malignancies. Specifically, this downstream signal transduction protein is involved in the activation of pathways required for B cell trafficking, chemotaxis, and adhesion, and has also been implicated in the initiation, survival, and progression of mature B cell lymphoproliferative disorders [2].

Ibrutinib is a selective BTK inhibitor that is administered orally. It was approved in 2014 for the treatment of chronic lymphocytic leukemia (CLL) and mantle cell lymphoma [3]. Ibrutinib acts by forming a covalent bond with cysteine-481 at the active site of BTK, irreversibly inhibiting BTK phosphorylation, which disrupts BCR signaling and interrupts B-lymphocyte proliferation and survival [4].

Ibrutinib is primarily metabolized by cytochrome P450 (CYP) CYP3A to yield a dihydrodiol metabolite with 15-fold lower BTK inhibitory activity than ibrutinib [3]. Absolute oral bioavailability is low, ranging from 3.9% to 8.4% depending on whether it is administered under fasting conditions or with standard breakfast, respectively [5]. Ibrutinib is rapidly absorbed following oral administration, with a time to peak concentration of 1–2 h. Its half-life is 4–13 h [3].

The CYP3A enzyme metabolizes about 30% of drugs and its functionality is mainly influenced by gender and induction and inhibition by multiple substances [6]. Multiple antifungal drugs, such as voriconazole, fluconazole, and posaconazole, that inhibit CYP3A4, can be administered in conjunction with ibrutinib to prevent or treat opportunistic infections. This coadministration could result in drug–drug interactions with ibrutinib and its metabolites through CYP3A. Related to CYP3A metabolizing enzymes, CYP3A4*20 (rs67666821) and CYP3A4*22 (rs35599367) polymorphisms have been associated with total and partial loss of enzyme function, respectively. CYP3A4*20 is found at a frequency of less than 0.1% in the European population. However, a frequency of up to 1.2% has been described in the Spanish population, due to a founder effect [7]. The frequency of the CYP3A4*22 allele is 5% in the European population. Moreover, CYP3A5*3 (rs776746) and CYP3A5*6 (rs10264272) alleles produce a truncated protein resulting in the absence of CYP3A5 [8] expression. These are present in the European population with a frequency of 94.3% and 0.3%, respectively.

It is essential to understand the pharmacokinetic profile of ibrutinib when it interacts with the CYP enzyme system, which can influence the drug’s metabolism and elimination, as well as its safety and efficacy profile [9].

The cytotoxic effects of ibrutinib were enhanced in vitro by the presence of inhibitors targeting breast cancer resistance protein, encoded by *ABCG2*, and P-glycoprotein, P-gp, encoded by *ABCB1* [10]. The *ABCB1* gene is highly polymorphic, as at least 38 variants have been described [11]. Among them, the most commonly studied variants are *ABCB1* rs1045642, rs1128503, and rs2032582, which affect P-gp functionality. While the impact of *ABCB1* polymorphisms on ibrutinib treatment has not been specifically addressed, the rs1045642, rs1128503, and rs2032582 variants were associated with clinical response to imatinib in chronic myeloid leukemia patients [12]. The wild-type ABCB1 protein appears to export imatinib more efficiently than the variant [12]. Interestingly, the impact of *ABCB1* polymorphisms on nilotinib and dasatinib seems to be less pronounced compared to imatinib [12]. However, the *ABCB1* 1199G>A polymorphism affected the transport activity of imatinib, nilotinib, and dasatinib, with the variant protein associated with lower antiproliferative effects [12]. It is believed that P-gp may play a role in the absorption, distribution, and excretion processes of multiple drugs [13,14]. Consequently, the expression level of P-gp and its functional integrity could influence the pharmacokinetics of drugs that are substrates of this transporter [15].

In vitro, ibrutinib was transported moderately by mouse Abcg2 but not detectably by human ABCG2 [16], so further approaches investigating the role of this transporters are needed. Finally, the solute carrier organic anion transporter, encoded by *SLCO1B1*, plays a significant role in hepatic drug uptake, which may affect systemic exposure. The *SLCO1B1* T521C polymorphism stands as a useful predictor for methotrexate-induced hepatotoxicity in patients with malignancies [17], but has never been explored in relation to ibrutinib treatment.

Ibrutinib is most commonly associated with diarrhea, upper respiratory tract infection, bleeding, fatigue, and heart-related adverse drug reactions (ADRs) [18]. These ADRs are typically mild (Common Terminology Criteria for Adverse Events CTCAE grade I–II). However, atrial fibrillation or bleeding can be life-threatening and must be strictly monitored. In fact, Mato et al. reported that 41% of 616 patients treated with ibrutinib in the United States withdrew ibrutinib (median time: 7 months), with toxicity being the most common reason for discontinuation [19]. Another study in 38 patients with relapsed CLL found an 81.6% frequency of ADRs associated with ibrutinib treatment and an 18% rate of treatment discontinuation due to ADRs [20].

Another study showed 303 cardiovascular deaths linked to ibrutinib, including those caused by supraventricular arrhythmias, CNS hemorrhages, CNS ischemia, heart failure, ventricular arrhythmias, and hypertension [21]. These cardiovascular ADRs were associated with a mortality rate ranging from 8% (for supraventricular and ventricular arrhythmias) to 20% (for CNS events and heart failure) [21]. In addition, ibrutinib-associated supraventricular arrhythmias foreshadow a poor prognosis when concurrent CNS events occur, resulting in a 28.8% mortality rate [21]. The researchers came to the conclusion that serious and occasionally fatal cardiac events occur in patients who were exposed to ibrutinib, and that these events, moreover, occur soon after the administration of ibrutinib [21]. There is a need for further strategies to minimize ibrutinib intolerance and maximize efficacy [19,20].

We intend to address interindividual variability in the efficacy and safety of CLL patients on ibrutinib treatment by quantifying plasma ibrutinib levels and determining *CYP3A* polymorphisms that reduce the functionality of the enzyme. There has not been enough discussion regarding the factors that lead to the discontinuation of treatment in clinical practice. Our study aims to shed light on this issue, as therapeutic drug monitoring (TDM) of ibrutinib could help determine whether plasma levels are within the therapeutic window or whether a dose adjustment is needed. In addition, a pharmacogenetic approach would be useful to identify the reason for interindividual variability in ibrutinib efficacy and the incidence of toxicity, as decreased enzyme activity and/or the use of concomitant medication would lead to decreased ibrutinib clearance and increased exposure. Therefore, determining the factors influencing ibrutinib treatment will help prevent ibrutinib-related toxicity.

We aim to address the variability in efficacy and safety among CLL patients undergoing ibrutinib treatment by quantifying plasma ibrutinib levels and identifying CYP3A polymorphisms that impair enzyme functionality. There has been insufficient discussion about the factors leading to treatment discontinuation in clinical practice. Our study seeks to illuminate this issue, as therapeutic drug monitoring (TDM) of ibrutinib could determine whether plasma levels fall within the therapeutic window or if a dose adjustment is necessary. Additionally, a pharmacogenetic approach would be beneficial in identifying the reasons for interindividual variability in ibrutinib efficacy and toxicity incidence, as reduced enzyme activity and/or the use of concomitant medication could result in decreased ibrutinib clearance and increased exposure. Therefore, identifying the factors influencing ibrutinib treatment will help prevent ibrutinib-related toxicity.

## 2. Results

A total of 49 patients were included: 22 patients from Hospital Universitario de Burgos (Burgos, Spain), 4 from Hospital Santiago Apóstol (Miranda de Ebro, Burgos, Spain), and 23 patients from Hospital Universitario de La Princesa (Madrid, Spain). Table 1 displays the most important demographic data for the population.

Seventy-one percent of the patients were men and twenty-nine were women with a mean age of 70 ± 9 years. The value on the Charlson Comorbidity Index (CCI) was greater than or equal to 5 in 78% of the patients. The main comorbidities presented by the patients were neoplasms (n = 10, 28%), diabetes (n = 5, 14%), chronic lung disease (n = 5, 14%), renal pathology (n = 4, 11%), mild liver disease (n = 3, 8%), peripheral vascular disease (n = 3, 8%), heart failure (n = 2, 6%), myocardial infarction (n = 1, 3%), solid metastasis (n = 1, 3%), malignant lymphomas (n = 1, 3%), and connective tissue pathologies (n = 1, 3%). The 10-year survival rate, as calculated using the CCI, had a median value of 21.36%.

Forty-nine percent of patients (n = 24) received ibrutinib as first-line treatment, and the remaining fifty-one percent (n = 25) received it due to resistance to other treatments.

### 2.1. Genotypic Frequencies

Genotyping was performed for the following genes: *ABCB1*, *ABCG2*, *CYP3A4*, *CYP3A5,* and *SLCO1B1*, finding the genotypic frequencies described in Table 2. The genotyping rate was higher than 93% for all the polymorphisms analyzed, except for CYP3A4*20, in which case genotyping was possible for 67.3% of the samples. The genotyping probe in this case was experimental and may have been the cause of this low genotyping rate. None of the successfully genotyped samples were carriers of the CYP3A4*20 allele.

All variants were found in Hardy–Weinberg equilibrium, with frequencies similar to those expected as described for the IBS population (Spanish population) in the 1000 Genomes Project [22]. No significant differences were observed in the genotype distribution between sexes.

### 2.2. Ibrutinib Plasma Concentrations

Of the 49 patients included, 8 were not sampled for plasma level measurement as they were no longer on ibrutinib treatment and their data collection was conducted entirely retrospectively. In addition, in 15 patients, the measurement result was below the lower limit of quantification (1 ng/mL), so a concentration of 0 ng/mL was considered. The mean trough concentration of ibrutinib was 1.77 ± 2.30 ng/mL.

### 2.3. Response Achieved

Patients were followed retrospectively and/or prospectively, with a median follow-up of 18.4 (2–104) months. Of the 45 patients with recorded response, 28 (62.2%) achieved CR, 11 (24.5%) achieved PR, 4 (8.9%) achieved Cri, and 2 (4.4%) showed NR.

No significant differences were found in the response achieved based on the sex of the subjects or whether the treatment was given as first or subsequent-line therapy.

### 2.4. Analysis of Toxicity

Seventy-six percent of the patients exhibited some form of toxicity, and 65 adverse drug reactions (ADRs) were documented, with causality assessed as possible, probable, or defined. The most frequent ADRs were vascular disorders (39%), including bleeding, followed by infections (27%), musculoskeletal disorders (10%), and nervous system disorders (10%), as shown in Table 3. One patient died due to complications from malignant prostate cancer, pulmonary metastases, and CLL.

The occurrence of ADRs showed no notable variations based on patient gender or whether the treatment was administered as first or subsequent-line therapy.

### 2.5. Influence of Genotypes on Ibrutinib Plasma Concentrations, Response, and ADRs

Regarding the association between genetic polymorphisms and ibrutinib levels, no significant differences were found, as observed in Table 4. Linear regression did not reveal any independent variables influencing plasma ibrutinib levels.

**Figure 1:**
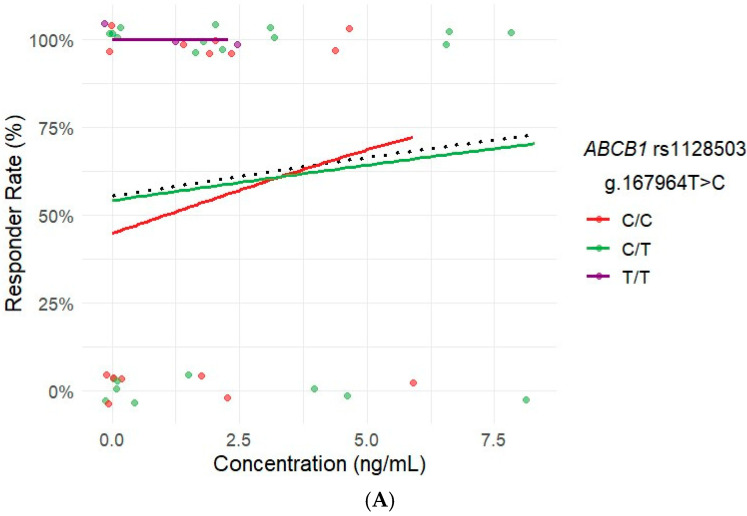

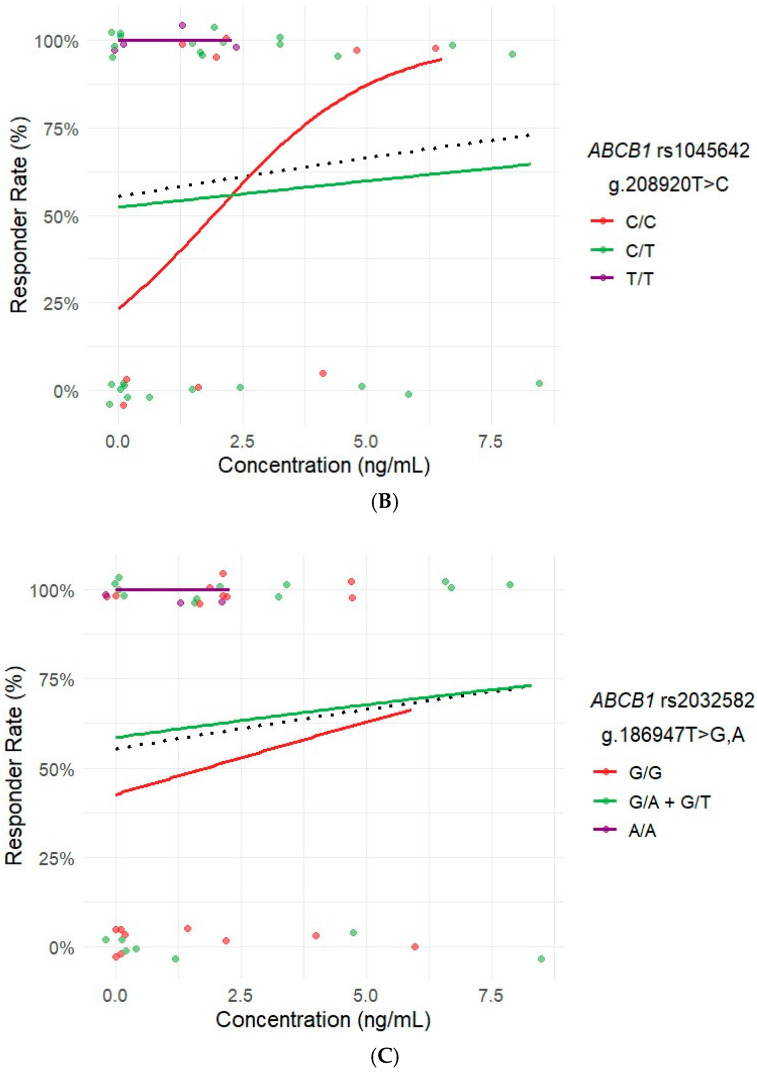
Impact of ibrutinib concentration on complete remission rate based on the different *ABCB1* rs1128503 (**A**), rs1045642 (**B**), and rs2032582 (**C**) genotypes: exposure–response analysis. The col-ored dots correspond to patients represented by genotype and concentration, while the colored curves show the model-predicted probabilities of achieving complete remission. The black dotted line represents the overall logistic regression trend across all genotypes, showing the general relationship between drug concentration and responder rate.

The influence of polymorphisms on the rate of CR was analyzed, finding no significant association for *CYP3A4*, *CYP3A5*, *ABCB1*, *ABCG2,* and *SLCO1B1* (Supplementary Figure S1). However, a tendency was observed for patients carrying *ABCB1* rs1128503 T/T (Figure 1A), rs1045642 T/T (Figure 1B), or rs2032582 A/A (Figure 1C) genotypes to achieve a higher CR rate.

In the univariate analysis, a significant association was found between CYP3A4*22 polymorphism and the incidence of overall ADRs, as one of the four CYP3A4*22 carriers showed an ADR, in contrast to the 90.5% of the wild-type carriers (*p* = 0.037) (Supplementary Table S1). However, no significant association was found between the remaining polymorphisms (*ABCB1*, *ABCG2*, *SLCO1B1*, and *CYP3A5*) and the incidence of ADRs overall, nor in the different subtypes of ADRs (Supplementary Tables S1–S5).

The findings of the multivariate analysis revealed that the incidence of overall ADRs was influenced by CYP3A4 functionality, with the *CYP3A4* *1/*22 genotype being a protective factor (OR = 0.071; CI95% 0.003–0.641). No other variables were found to be significant.

### 2.6. Influence of Ibrutinib Plasma Concentrations on Response and ADRs

The study revealed no statistically significant correlation between the concentration of ibrutinib and the rate of CR (*p* = 0.577). Additionally, as observed in the exposure–response analysis, the dispersion of the data suggests that other factors may be influencing the treatment’s efficacy (Supplementary Figure S2). Similarly, there were no significant differences between patients who developed overall ADRs (1.63 ± 2.24 ng/mL) and those who did not (2.21 ± 2.51 ng/mL), *p* = 0.455. There were also no significant differences in any subtype of ADRs.

### 2.7. Influence of Concomitant Medication on Response and ADRs

Among the included patients, 14 (28.6%) were concomitantly administered CYP3A4 inducers (prednisone, dexamethasone, metamizole, budesonide, terbinafine, or mometasone), while 17 patients (34.7%) received a CYP3A4 inhibitor (omeprazole, ciprofloxacin).

In relation to ibrutinib’s efficacy, neither the concomitant administration of CYP3A4 inducers nor that of CYP3A4 inhibitors significantly affected the final response achieved (*p*= 0.7 in both cases).

Regarding toxicity, across both univariate and multivariate analyses, no statistically significant associations were observed between the use of CYP3A4 inducers or inhibitors and the incidence of ADRs (Supplementary Tables S6 and S7).

## 3. Discussion

Ibrutinib, an antineoplastic agent for CLL, is characterized by a combination of low solubility and high permeability. Its oral bioavailability remains a perplexing challenge, merely reaching 2.9% due to formidable first-pass metabolism hurdles, which include high liver enzyme activity, particularly CYP3A4, and variable metabolism rates, influenced by genetic differences and drug interactions [23].

Ibrutinib has shown remarkable effectiveness in treating CLL. In our study, the majority of patients responded to treatment, with 62% achieving CR and 9% achieving CRi. Additionally, 24% of patients achieved PR, and only 4.4% of patients showed NR. These results are comparable to those obtained in the RESONATE-2 clinical trial. According to Barr et al. [24], the RESONATE-2 trial demonstrated high efficacy of ibrutinib in the treatment of CLL among older patients. The study reported an overall response rate of 92%, with 18% of patients achieving CR or CRi. The progression-free survival rate at 24 months was 89%, and the overall survival rate at 24 months was 95% [24]. Our study’s higher CR/CRi rate (71% combined) compared to RESONATE-2 (18%) may be due to differences in patient populations, treatment duration, or assessment criteria. Nevertheless, both studies consistently highlight ibrutinib’s strong performance in CLL treatment, with most patients showing favorable responses to the therapy.

Ibrutinib is generally well-tolerated. Specifically, it is one of the agents that has shown excellent activity in CLL, particularly in cases with 17p deletions, where traditional therapies often fail [25]. However, side effects such as atrial fibrillation and bleeding, which can be severe, occur [18]. Grade III or higher side effects require strict monitoring. The most common side effects, which are generally mild, include diarrhea, upper respiratory tract infections, bleeding, fatigue, and heart-related ADRs [18]. In fact, a study published in June 2020 revealed that toxicity was the most common reason for discontinuing ibrutinib treatment in routine clinical practice [26]. Our study showed that among the recorded ADRs, the most frequent ones were vascular disorders (39%), including hemorrhages, followed by infections (27%), and musculoskeletal (10%) and neurological (10%) disorders. Mato et al. reported that among the patients treated front-line with ibrutinib, the three most common toxicities leading to discontinuation were arthralgia (41.6%), atrial fibrillation (25%), and rash (16.7%); while in the relapsed/refractory population, the most common toxicities leading to discontinuation were atrial fibrillation (12.3%), infection (10.7%), pneumonitis (9.9%), bleeding (9%), and diarrhea (6.6%) [19]. Variations in comorbidities or pre-existing conditions, such as cardiovascular risk factors, could explain why vascular disorders dominate our ADRs, whereas atrial fibrillation appears more frequently in Mato et al.’s cohorts.

The observed association between the *CYP3A4* *1/*22 genotype and a lower incidence of ADRs contradicts the expected outcome, given that CYP3A4 is the primary enzyme responsible for ibrutinib metabolism, and that CYP3A4*22 polymorphism is associated with reduced hepatic CYP3A4 activity [27,28]. A decreased metabolic capacity would typically result in lower drug clearance and increased exposure, potentially leading to a higher incidence of ADRs. However, our findings align with previous reports suggesting that *CYP3A4* polymorphisms may have a limited influence on the pharmacokinetics of some drugs primarily metabolized by this enzyme, likely due to its high inducibility and compensatory mechanisms, especially due to the majority of subjects lacking the CYP3A5 enzyme [29]. Furthermore, the impact of *CYP3A4* variants may only be significant in a small subset of *CYP3A4* poor metabolizer patients [29]. In heterozygous individuals, the effect may be less pronounced due to the inducibility of the CYP3A4 enzyme. Given the small number of CYP3A4*22 carriers in our cohort, these results should be interpreted with caution. Larger studies are needed to elucidate the precise role of *CYP3A4* polymorphisms in ibrutinib metabolism and their potential clinical implications.

Related to drug transporters, our analysis revealed a trend suggesting that patients with *ABCB1* rs1128503, rs1045642 T/T, or rs2032582 A/A genotypes achieved a higher response rate to treatment. This finding is consistent with the observed trend of a lower incidence of ADRs in this patient subgroup. Polymorphisms in *ABCB1* may alter transporter function, potentially enhancing intracellular drug accumulation and efficacy while simultaneously reducing off-target effects that contribute to ADRs. Although these findings are promising, it is important to interpret them with caution given the limited sample size.

Hardy-Abeloos et al. observed that patients receiving concomitant treatment with CYP3A4 inhibitors should receive lower doses than the standard 420 mg daily [26]. In our study, although 17 patients received CYP3A4 inhibitors, 16 of them were treated with omeprazole, which is considered a weak CYP3A4 inhibitor. While omeprazole does interact with CYP3A4 to some extent, it primarily acts as an inhibitor of CYP2C19 [30]. This may have limited the likelihood of detecting a strong pharmacokinetic interaction requiring dose adjustment. Accordingly, we did not observe any statistically significant differences in treatment efficacy or toxicity among these patients. Further prospective studies with larger patient cohorts are required to validate the need for dose adjustments and to inform evidence-based clinical guidelines in this context.

Having more robust ibrutinib trough concentration data would have added significant value to the analysis; however, the scarcity of evaluable samples has hindered this possibility. The exclusion of eight patients from plasma level measurement due to retrospective data collection, as well as the fact that fifteen patients had concentrations below the lower limit of quantification, significantly reduced the number of evaluable samples. This, combined with the high variability in trough concentrations (mean 1.77 ± 2.30 ng/mL), may have influenced the lack of significant associations observed. These results limit the ability to draw more objective conclusions regarding the relationship between ibrutinib plasma levels, ADRs, and genetic polymorphisms.

### Study Limitations

Although candidate gene studies have proven to be an effective strategy for identifying polymorphisms associated with different pharmacological responses, there is a potential risk of bias in the selection of genes and polymorphisms to be analyzed. As a result, it is possible that some less frequent polymorphisms could influence the response to ibrutinib treatment, but we may be unable to identify them due to the low number of subjects carrying these polymorphisms. Additionally, the small sample size limited us from finding more patients carrying some minor alleles with a low frequency that might be related to ibrutinib metabolism; as well as a low frequency of certain events, which complicates statistical analysis and the ability to draw definitive conclusions. Although CLL is not an extremely rare disease, it remains a low-prevalence condition in the general population, which can pose challenges for conducting large-scale studies, particularly in specific patient subgroups such as those treated with ibrutinib. This limitation highlights the need for multicenter collaborations and larger cohorts to obtain robust conclusions regarding the pharmacogenetic and clinical implications of ibrutinib therapy. Another limitation of this study is the use of only trough plasma concentrations, which does not allow for full pharmacokinetic profiling. This restricts the evaluation of parameters such as AUC or variability in drug levels. Future studies should consider more comprehensive sampling to better capture interindividual differences in exposure. Lastly, another notable limitation is the study design, as some patients were included retrospectively. While such studies can provide valuable information about associations between variables, their results should be interpreted with caution due to inherent limitations, including the risk of selection, information, and confounding biases, as well as the difficulty in establishing cause-and-effect relationships. Potential residual bias arising from the combination of retrospective and prospective data collection cannot be completely excluded, despite the application of consistent inclusion criteria and standardized procedures.

## 4. Materials and Methods

### 4.1. Study Population, Design, and Procedures

This is an observational, retrospective, and/or prospective follow-up study in a cohort of CLL patients treated with ibrutinib. Patient selection and follow-up was carried out by the Hematology Department and the Research Unit of the Hospital Universitario de Burgos (HUBU), Burgos (Spain), and by the Hematology Department and the Clinical Pharmacology Department of the Hospital Universitario de La Princesa (HULP), Madrid (Spain). This is a pragmatic study intended to demonstrate the usefulness of pharmacogenetics in routine clinical practice, so the usual procedures for this type of patient were not modified. Routinely scheduled visits were used to collect the necessary samples and data. Patients who had previously received ibrutinib were tracked retrospectively from the beginning of treatment to the end of the study (February 2023) or treatment interruption and discontinuation. Prospectively enrolled patients were followed for a minimum of 6 months or until treatment was discontinued.

The primary safety endpoint was the incidence and severity of ADRs. To assess safety, the incidence of adverse events was collected and tabulated by organ-system class based on ibrutinib drug label [3]. Death from any cause was also recorded. Causality of identified adverse events was determined using the causality algorithm of the Spanish Pharmacovigilance System [27]. Only those categorized as definite, probable, or possible adverse events were considered as ADRs and included in the statistical analysis. Intensity and outcome of ADRs were also recorded.

Moreover, assessment of treatment response as complete remission (CR), complete remission with incomplete hematological recovery (CRi), partial response (PR), no response (NR), relapsed (RE), and exitus (EX) was collected. The response to treatment was assessed according to the criteria of the Spanish Group of Chronic Lymphocytic Leukemia (GELLC), as detailed in their national clinical practice guidelines [31].

All information was obtained from the medical records and contained demographic factors (age, sex, ethnicity), body mass index (BMI), concomitant medication and comorbidities, as well as Charlson’s Comorbidity Index (CCI) [28]. Concomitant treatment with CYP3A4 inhibitors and inducers was also taken into account.

### 4.2. Genotyping

A MagNA Pure LC DNA Isolation Kit was used in a MagNa Pure^®^ System (Roche Applied Science, Indianapolis, IN, USA) automatic DNA extractor to isolate DNA from 1 mL of peripheral blood samples. The A_260/280_ absorbance ratio was used to quantitatively determine sample purity using a NanoDrop^®^ ND-1000 Spectrophotometer (NanoDrop Technologies Inc., Wilmington, DE, USA).

In this study, we looked at 9 polymorphisms in 5 genes involved in the metabolism and transport of ibrutinib. A complete list of the analyzed variants and their functional consequences is described in Table 5. The genotyping was performed by quantitative polymerase chain reaction (qPCR) using a ViiA7^®^ PCR Instrument (Applied Biosystems, Foster City, CA, USA) and TaqMan assays following the manufacturer recommendations (Applied Biosystems, Foster City, CA, USA). All assays were performed with an internal quality control, with a reproducibility of 100%.

### 4.3. Measurement of Ibrutinib Plasma Concentrations

After at least 7 days of ibrutinib treatment and right before the next dose, a blood sample was obtained for the analysis of ibrutinib trough plasma concentrations. Samples were centrifuged at 4 °C for 10 min at 3500 rpm (1900× *g*). All plasma samples were stored at −80 °C ± 5 °C until their shipment to the Analytical Laboratory of Clinical Pharmacology Department of Hospital Universitario de La Princesa, where ibrutinib plasma concentrations were determined using high performance liquid chromatography coupled to a tandem mass spectrometry detector (HPLC-MS/MS). The measurement method is already validated according to EMA, FDA and ICH guidelines, using a 1290 HPLC instrument and an Agilent 6410 triple quadrupole mass spectrometer (Agilent, Santa Clara, CA, USA) [32]. The method established a lower limit of quantification of ibrutinib of 1 ng/mL.

Ibrutinib’s half-life is estimated to be 4–13 h [3]. Assuming an intermediate elimination half-life of 9 h, 95% of the ibrutinib concentration will have been eliminated in 5 half-lives (45 h). For the calculation of the elimination constant, an exponential regression was performed since the drug has an elimination kinetics of order 1, which means that the elimination rate depends on the previous concentration, which is not a constant concentration. Taking into account the time of extraction of the sample and its concentration, the trough concentration was calculated according to the formula C = Co*e^-kt, where k is the calculated elimination constant (in this case 0.067), Co corresponds to the concentration of the sample at t = 0 (time of extraction), and t corresponds to the time to the exact trough for each sample. This study was conducted as an observational investigation within the context of routine clinical practice, where the collection of multiple time points per patient was not feasible due to logistical and ethical constraints. As a result, trough concentrations were selected as the most practical and reproducible pharmacokinetic marker, particularly due to their correlation with steady-state exposure and potential toxicity [33].

### 4.4. Statistical Analysis

A descriptive and frequency analysis was performed, as well as univariate and multivariate analysis, with *p*-values <0.05 being considered significant. The main variable was the incidence of ADRs. The relationship of this variable with the following possible factors was evaluated: age, sex, weight, BMI, race, concomitant treatment, comorbidities, response achieved, and genetic polymorphisms. Differences in genotype frequencies according to sex and the comparison of the qualitative variables between different genotypes were determined using a corrected Pearson chi-square test and Fisher’s exact test. Differences in quantitative parameters between individuals were statistically analyzed by a parametric univariate analysis (*t* test or ANOVA) or non-parametric univariate analysis (Kruskal–Wallis). Multivariate analyses were performed using step-wise logistic regression to investigate the effect on response rate and incidence of ADRs, and a multiple linear regression analysis to study the effect on plasma levels of ibrutinib. *p*-Values were adjusted for multiple testing using the Benjamini–Hochberg procedure to control the false discovery rate (FDR). All demographic and clinical variables relevant to the outcome, as well as the different genotypes, were included as independent variables. Patients were censored when either ibrutinib treatment or the follow-up was finished. All these analyses were performed in the Research Unit of the Hospital Universitario de Burgos using R statistical software, version 4.1.1.

## 5. Conclusions

This study provides novel insights into the pharmacogenetic and clinical factors influencing ibrutinib therapy in patients with CLL. Our findings suggest that the CYP3A4*22 variant may be associated with a lower incidence of ADRs, potentially reflecting compensatory metabolic pathways that mitigate the impact of reduced CYP3A4 activity. Although this result contradicts initial expectations, it aligns with prior reports and warrants confirmation in larger cohorts.

We also observed a trend toward improved treatment response and fewer ADRs in patients carrying certain *ABCB1* genotypes, supporting the hypothesis that drug transport mechanisms may influence both efficacy and tolerability.

From a clinical perspective, these findings underscore the potential utility of incorporating *CYP3A4* and *ABCB1* genotyping into treatment planning. For translational research, our results point to the need for further exploration of alternative metabolic and transport pathways involved in ibrutinib disposition. Larger, prospective studies integrating pharmacokinetic profiling and pharmacogenetic data will be essential to validate these associations and support more personalized approaches to ibrutinib therapy in CLL. Finally, efforts to shed light on drug–drug interactions that may reduce ibrutinib exposure, as well as to identify additional non-genetic factors, will be essential for achieving a deeper understanding of the interindividual variability in treatment response and toxicity.

## Figures and Tables

**Table 1:** Demographic and clinical characteristics of the study population.

Variable	N (%) or Mean (SD)
Sex	
Men	35 (71)
Women	14 (29)
Age	70 (9)
Weight	72 (13.0)
Height	1.67 (0.08)
BMI	26.5 (3.6)
CCI score (median)	5 (3–15)
10-year survival (median of CCI calculated percentage)	21.36 (0–77.48)
Line of therapy	
First-line therapy	24 (49)
≥Second-line therapy	25 (51)

Abbreviation: BMI, body mass index; CCI, Charlson Comorbidity Index; SD, standard deviation.

**Table 2:** Genotypic frequencies of the genes analyzed according to sex.

Gene	Genotype	Total Frequency (n = 49) N (%)	Frequency in Men (n = 35) N (%)	Frequency in Women (n = 14) N (%)	*p*-Value
*ABCB1* rs1045642 g.208920T>C	C/C	11 (22.9)	8 (23.5)	3 (21.4)	0.5
	C/T	31 (64.6)	23 (67.7)	8 (57.2)	
	T/T	6 (12.5)	3 (8.8)	3 (21.4)	
*ABCB1* rs1128503 g.167964T>C	C/C	17 (35.4)	14 (41.2)	3 (21.4)	0.062
	C/T	27 (56.3)	19 (55.9)	8 (57.2)	
	T/T	4 (8.3)	1 (2.9)	3 (21.4)	
*ABCB1* rs2032582 g.186947T>G,A	G/G	19 (39.6)	15 (44.1)	4 (28.6)	0.12
	G/A + G/T	25 (52.1)	18 (53.0)	7 (50.0)	
	A/A	4 (8.3)	1 (2.9)	3 (21.4)	
*ABCG2* rs2231137 g.89061114C>T	C/C	44 (91.7)	30 (88.2)	14 (100.0)	0.3
	C/T	4 (8.3)	4 (11.8)	0	
*ABCG2* rs2231142 g.89052323G>T	G/G	43 (91.5)	31 (93.9)	12 (85.7)	0.6
	G/T	4 (8.5)	2 (6.1)	2 (14.3)	
*SLCO1B1* rs4149056 g.21331549T>C	T/T	35 (72.9)	25 (73.5)	10 (71.4)	>0.9
	C/T	13 (27.1)	9 (26.5)	4 (28.6)	
*CYP3A4*	*1/*1	42 (91.3)	28 (87.5)	14 (100.0)	0.3
	*1/*22	4 (8.7)	4 (12.5)	0	
*CYP3A5*	*1/*3	2 (4.3)	1 (3.0)	1 (7.2)	0.5
	*3/*3	45 (95.7)	32 (97.0)	13 (92.8)	

**Table 3:** Frequency of ADRs according to sex.

Type of Adverse Reaction	Total Frequency (n = 49) N (%)	Frequency in Men (n = 35) N (%)	Frequency in Women (n = 14) N (%)	*p*-Value
Infections and infestations	13 (26.5)	11 (31.4)	2 (14.3)	0.3
Neoplasms	4 (8.2)	4 (11.4)	0	0.3
Nervous system disorders	5 (10.2)	4 (11.4)	1 (7.1)	>0.9
Cardiac disorders	3 (6.1)	2 (5.7)	1 (7.1)	>0.9
Skin and subcutaneous tissue disorders	2 (4.1)	2 (5.7)	0	>0.9
Blood and lymphatic system disorders	4 (8.2)	2 (5.7)	2 (14.3)	0.6
Metabolism and nutritional disorders	3 (6.1)	2 (5.7)	1 (7.1)	>0.9
Gastrointestinal disorders	4 (8.2)	1 (2.9)	3 (21.4)	0.065
Hepatobiliary disorders	1 (2.0)	1 (2.9)	0	>0.9
Musculoskeletal disorders	5 (10.2)	4 (11.4)	1 (7.1)	>0.9
Vascular disorders	19 (38.8)	13 (37.1)	6 (42.9)	0.7
General disorders	2 (4.1)	2 (5.7)	0	>0.9

**Table 4:** Plasma levels of ibrutinib based on the different genotypes.

Gene	Genotype	Mean Ibrutinib Concentration (ng/mL)	SD	*p*-Value
*ABCB1* rs1045642 g.208920T>C	C/C (n = 11)	2.50	2.17	0.527
	C/T (n = 31)	2.14	2.58	
	T/T (n = 6)	0.87	1.10	
*ABCB1* rs1128503 g.167964T>C	C/C (n = 17)	1.77	1.94	0.560
	C/T (n = 27)	2.44	2.76	
	T/T (n = 4)	1.16	1.14	
*ABCB1* rs2032582 g.186947T>G,A	G/G (n = 19)	1.82	1.90	0.546
	G/A + G/T (n = 25)	2.50	2.90	
	A/A (n = 4)	1.16	1.14	
*ABCG2* rs2231137 g.89061114C>T	C/C (n = 44)	2.11	2.35	0.996
	C/T (n = 4)	2.16	3.16	
*ABCG2* rs2231142 g.89052323G>T	G/G (n = 43)	2.30	2.43	0.249
	G/T (n = 4)	0.828	1.66	
*SLCO1B1* rs4149056 g.21331549T>C	C/T (n = 13)	2.48	1.74	0.615
	T/T (n = 35)	2.00	2.54	
*CYP3A4*	*1/*1 (n = 42)	2.08	2.35	0.287
	*1/*22 (n = 4)	3.29	2.88	
*CYP3A5*	*1/*3 (n = 0)	NA	NA	NA *
	*3/*3 (n = 25)	2.15	2.39	

Abbreviation: SD, standard deviation. * Note: For the *CYP3A5* polymorphism, all individuals with available pharmacokinetic measurements carried the *3/3 genotype. Therefore, the model cannot estimate group differences, and the analysis is not applicable, so no *p*-value is reported for this variant.

**Table 5:** Genes and variants analyzed involved in the metabolism and transport of ibrutinib.

Gene	Variant	rs Number	Reference	RefSeq Reference Allele	RefSeq Alternative Allele	Consequence	MAF in European Population
*ABCB1*	g.167964T>C	rs1128503	C___7586662_10	A	G	Synonymous variant	0.584
*ABCB1*	g.208920T>C	rs1045642	C___7586657_20	A	G	Missense Variant	0.482
*ABCB1*	g.186947T>G,A	rs2032582	C_11711720C_30	A	G	Missense Variant	0.573
*ABCB1*	g.186947T>G,A	rs2032582	C_11711720D_40	A	T	Missense Variant	0.001
*ABCG2*	g.89061114C>T	rs2231137	ANXG6CY	C	T	Missense Variant	0.16
*ABCG2*	g.89052323G>T	rs2231142	C__15854163_70	G	T	Stop Gained	0.12
*CYP3A4*	*20	rs67666821	ANNKRXD	TTTTT	TTTTTT	Frameshift Variant	0.0001
*CYP3A4*	*22	rs35599367	C__59013445_10	G	A	Intron Variant	0.05
*CYP3A5*	*3	rs776746	C__26201809_30	T	C	Splice Acceptor Variant	0.943
*SLCO1B1*	g.21331549T>C	rs4149056	C__30633906_10	T	C	Missense Variant	0.22

Abbreviation: *ABCB1*, ATP binding cassette subfamily B member 1; *ABCG2*, ATP binding cassette subfamily G member 2; *CYP3A4*, cytochrome P450 family 3 subfamily A member 4; *CYP3A5*, cytochrome P450 family 3 subfamily A member 5; *SLCO1B1*, solute carrier organic anion transporter family member 1B1; MAF, minor allele frequency; RefSeq, Reference Sequence collection.

## Data Availability

The original contributions presented in the study are included in the article and supplementary material; further inquiries can be directed to the corresponding author.

## Supplementary Materials

The following supporting information can be downloaded at: https://www.mdpi.com/article/10.3390/ph18070996/s1, Supplementary Figure S1: Impact of ibrutinib concentration on complete remission rate based on the different *ABCG2*, *SLCO1B1* and *CYP3A4* genotypes: exposure-response analysis; Supplementary Figure S2: Impact of ibrutinib concentration on complete remission rate: exposure-response analysis; Supplementary Table S1: Incidence of overall ADRs based on the genotype; Supplementary Table S2: Incidence of infections and infestations, neoplasms and nervous system disorders based on the genotype; Supplementary Table S3: Incidence of cardiac, skin and subcutaneous tissue, and blood and lymphatic system disorders based on the genotype; Supplementary Table S4: Incidence of metabolism and nutritional, gastrointestinal and hepatobiliary disorders based on the genotype; Supplementary Table S5: Incidence of musculoskeletal, vascular and general disorders based on the genotype; Supplementary Table S6: Influence of concomitant administration of CYP3A4-inducers on ADRs; Supplementary Table S7: Influence of concomitant administration of CYP3A4-inhibitors on ADRs.
