# Supplementary material for: Pharmacogenetic Biomarkers of Ibrutinib Response and Toxicity in Chronic Lymphocytic Leukemia: Insights from an Observational Study
Source: Pharmaceuticals (Basel). 2025 Jul 2;18(7):996. doi: 10.3390/ph18070996 (PMC12299598; doi:10.3390/ph18070996)
Supplement: Supplementary file 1 [file pharmaceuticals-18-00996-s001.zip › pharmaceuticals-3688648-supplementary.pdf]

Supplementary Materials

A)

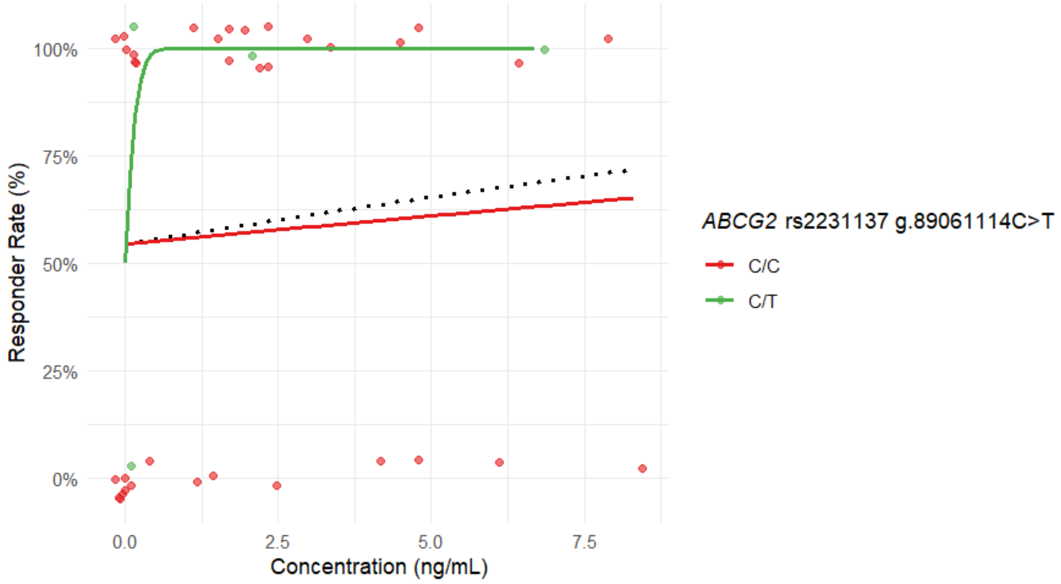

B)

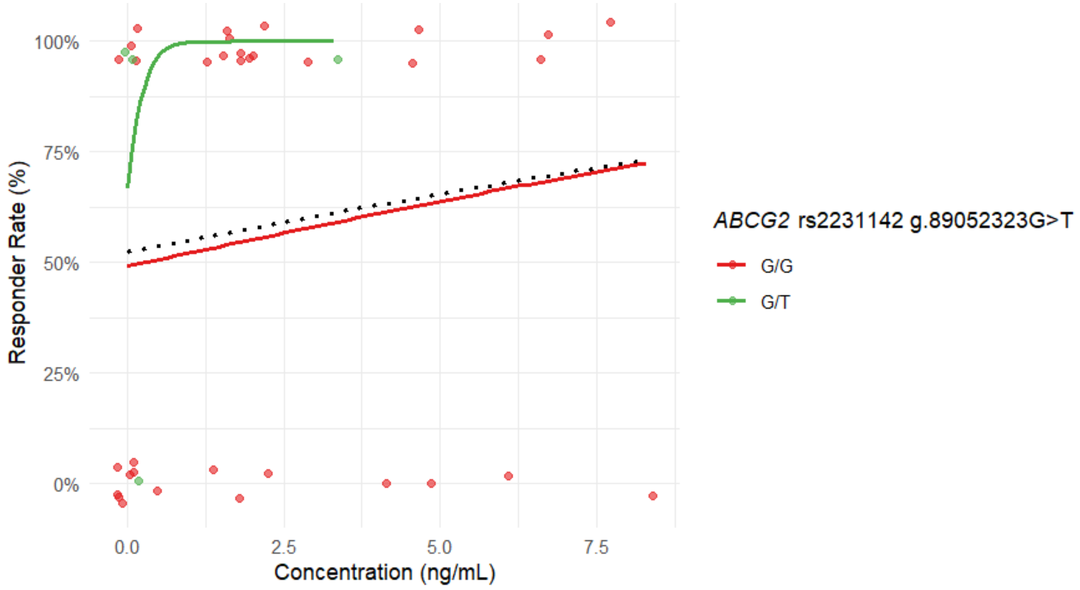

C)

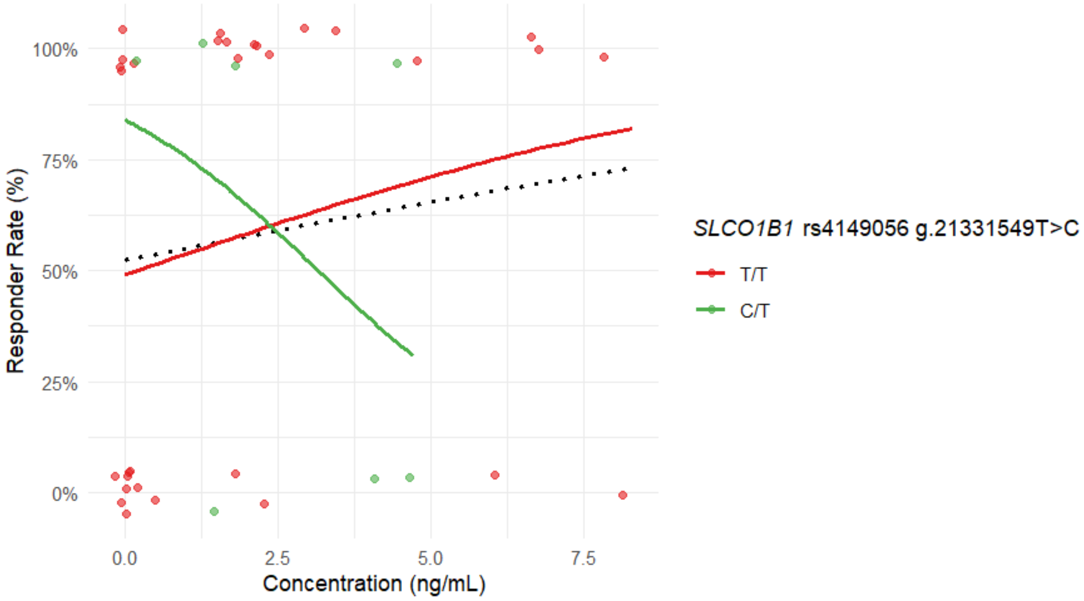

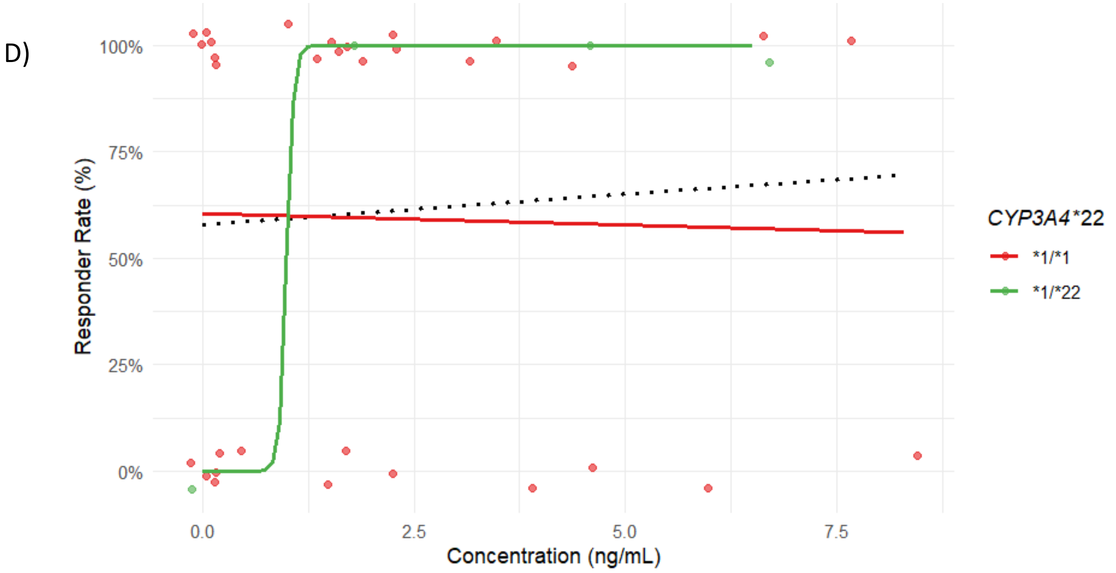

Supplementary Figure S1. Impact of ibrutinib concentration on complete remission rate based on the different *ABCG2*, *SLCO1B1* and *CYP3A4* genotypes: exposure-response analysis. The coloured dots correspond to patients represented by genotype and concentration, while the coloured curves show the model-predicted probabilities of achieving complete remission.

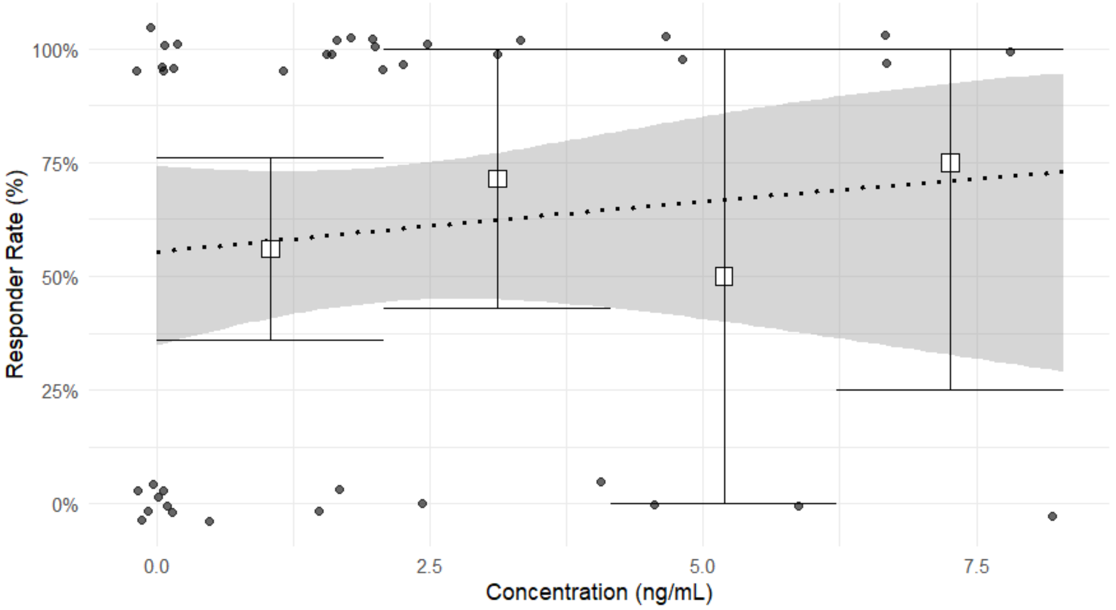

Supplementary Figure S2. Impact of ibrutinib concentration on complete remission rate: exposure-response analysis. Solid white squares represent the proportion of responders grouped by quartiles of concentration and plotted at the median concentration for each quartile. Vertical lines represent the 90% confidence interval for each quartile. The black curve and shaded grey area represent model-predicted probabilities and their 95% confidence interval, respectively. The analysis included 45 patients with recorded response data out of a total of 49, as four patients had missing response records. The exclusion of eight patients from plasma level measurement due to retrospective data collection, along with 15 patients having concentrations below the lower limit of quantification, significantly reduced the number of evaluable samples.

Supplementary Table S1. Incidence of overall ADRs based on the genotype.

	Overall Adverse Drug Reactions		
**Genotype**	**No**<br>N = 12[1]	**Yes**<br>N = 36[1]	***p*-value**[2]
ABCB1 rs1045642 g.208920T>C			0.8
C/C	3 (27%)	8 (73%)	
C/T	7 (23%)	24 (77%)	
T/T	2 (33%)	4 (67%)	
ABCB1 rs1128503 g.167964T>C			0.5
C/C	4 (24%)	13 (76%)	
C/T	6 (22%)	21 (78%)	
T/T	2 (50%)	2 (50%)	
ABCB1 rs2032582 g.186947T>G,A			0.4
A/A	2 (50%)	2 (50%)	
G/A + G/T	6 (24%)	19 (76%)	
G/G	4 (21%)	15 (79%)	
ABCG2 rs2231137 g.89061114C>T			0.3
C/C	10 (23%)	34 (77%)	
C/T	2 (50%)	2 (50%)	
ABCG2 rs2231142 g.89052323G>T			>0.9
G/G	10 (23%)	33 (77%)	
G/T	1 (25%)	3 (75%)	
SLCO1B1 rs4149056 g.21331549T>C			0.14
C/T	1 (7.7%)	12 (92%)	
T/T	11 (31%)	24 (69%)	
CYP3A4			0.037
*1/*1	8 (19%)	34 (81%)	
*1/*22	3 (75%)	1 (25%)	
CYP3A5			>0.9
*1/*3	0 (0%)	2 (100%)	
*3/*3	11 (24%)	34 (76%)	

[1] n (%), [2] Fisher's exact test.

Supplementary Table S2. Incidence of infections and infestations, neoplasms and nervous system disorders based on the genotype.

	**Infections and infestations**			**Neoplasms**			**Nervous system disorders**		
**Genotype**	**No** N = 35[1]	**Yes** N = 13[1]	***p*-value**[2]	**No** N = 38[1]	**Yes** N = 10[1]	***p*-value**[2]	**No** N = 44[1]	**Yes** N = 4[1]	***p*-value**[2]
ABCB1 rs1045642 g.208920T>C			0.9			0.14			0.6
C/C	8 (72.7%)	3 (27.3%)		7 (63.7%)	4 (36.3%)		10 (90.9%)	1 (9.1%)	
C/T	23 (74.2%)	8 (25.8%)		27 (87.1%)	4 (12.9%)		29 (93.5%)	2 (6.5%)	
T/T	4 (66.7%)	2 (33.3%)		4 (66.7%)	2 (33.3%)		5 (83.3%)	1 (16.7%)	
ABCB1 rs1128503 g.167964T>C			0.4			0.8			>0.9
C/C	11 (64.7%)	6 (35.3%)		13 (76.5%)	4 (23.5%)		16 (94.1%)	1 (5.9%)	
C/T	20 (74.1%)	7 (25.9%)		21 (77.8%)	6 (22.2%)		24 (88.9%)	3 (11.1%)	
T/T	4 (100.0%)	0		4 (100.0%)	0		4 (100.0%)	0	
ABCB1 rs2032582 g.186947T>G,A			0.4			0.7			>0.9
G/G	12 (63.2%)	7 (36.8%)		14 (73.7%)	5 (26.3%)		17 (89.5%)	2 (10.5%)	
G/A + G/T	19 (76.0%)	6 (24.0%)		20 (80.0%)	5 (20.0%)		23 (92.0%)	2 (8.0%)	
A/A	4 (100.0%)	0		4 (100.0%)	0		4 (100.0%)	0	
ABCG2 rs2231137 g.89061114C>T			0.6			0.2			0.4
C/C	31 (70.5%)	13 (29.5%)		36 (81.8%)	8 (18.2%)		40 (90.9%)	4 (9.1%)	
C/T	4 (100.0%)	0		2 (50.0%)	2 (50.0%)		3 (75.0%)	1 (25.0%)	
ABCG2 rs2231142 g.89052323G>T			>0.9			0.6			0.3
G/G	31 (72.1%)	12 (27.9%)		33 (76.7%)	10 (23.3%)		40 (93.0%)	3 (7.0%)	
G/T	3 (75.0%)	1 (25.0%)		4 (100.0%)	0		3 (75.0%)	1 (25.0%)	
SLCO1B1 rs4149056 g.21331549T>C			0.3			0.7			0.6
C/T	8 (61.5%)	5 (38.5%)		11 (84.6%)	2 (15.4%)		13 (100.0%)	0	
T/T	27 (77.1%)	8 (22.9%)		27 (77.1%)	8 (22.9%)		31 (88.6%)	4 (11.4%)	
CYP3A4			0.6			>0.9			0.3
*1/*1	30 (71.4%)	12 (28.6%)		34 (81.0%)	8 (19.0%)		39 (92.9%)	3 (7.1%)	
*1/*22	4 (100.0%)	0		3 (75.0%)	1 (25.0%)		3 (75.0%)	1 (25.0%)	
CYP3A5			0.5			0.3			>0.9
*1/*3	1 (50.0%)	1 (50.0%)		1 (50.0%)	1 (50.0%)		2 (100.0%)	0	
*3/*3	33 (73.3%)	12 (26.7%)		37 (82.2%)	8 (17.8%)		41 (91.1%)	4 (8.9%)	

[1] n (%), [2] Fisher's exact test.

Supplementary Table S3. Incidence of cardiac, skin and subcutaneous tissue, and blood and lymphatic system disorders based on the genotype.

	Cardiac disorders			Skin and subcutaneous tissue disorders			Blood and lymphatic system disorders		
**Genotype**	**No** N = 45[1]	**Yes** N = 3[1]	***p*-value**[2]	**No** N = 46[1]	**Yes** N = 2[1]	***p*-value**[2]	**No** N = 44[1]	**Yes** N = 4[1]	***p*-value**[2]
ABCB1 rs1045642 g.208920T>C			0.7			>0.9			0.7
C/C	11 (100.0%)	0		11 (100.0%)	0		11 (100.0%)	0	
C/T	28 (90.3%)	3 (9.7%)		29 (93.5%)	2 (6.5%)		27 (87.1%)	4 (12.9%)	
T/T	6 (100.0%)	0		6 (100.0%)	0		6 (100.0%)	0	
ABCB1 rs1128503 g.167964T>C			0.4			0.6			0.3
C/C	17 (100.0%)	0		17 (100.0%)	0		17 (100.0%)	0	
C/T	24 (88.9%)	3 (11.1%)		25 (92.6%)	2 (7.4%)		23 (85.2%)	4 (14.8%)	
T/T	4 (100.0%)	0		4 (100.0%)	0		4 (100.0%)	0	
ABCB1 rs2032582 g.186947T>G,A			0.7			>0.9			0.2
G/G	17 (89.5%)	2 (10.5%)		18 (94.7%)	1 (5.3%)		19 (100.0%)	0	
G/A + G/T	24 (96.0%)	1 (4.0%)		24 (96.0%)	1 (4.0%)		21 (84.0%)	4 (16.0%)	
A/A	4 (100.0%)	0		4 (100.0%)	0		4 (100.0%)	0	
ABCG2 rs2231137 g.89061114C>T			>0.9			>0.9			>0.9
C/C	41 (93.2%)	3 (6.8%)		42 (95.5%)	2 (4.5%)		40 (90.9%)	4 (9.1%)	
C/T	4 (100.0%)	0		4 (100.0%)	0		4 (100.0%)	0	
ABCG2 rs2231142 g.89052323G>T			>0.9			>0.9			>0.9
G/G	40 (93.0%)	3 (7.0%)		41 (95.3%)	2 (4.7%)		39 (90.7%)	4 (9.3%)	
G/T	4 (100.0%)	0		4 (100.0%)	0		4 (100.0%)	0	
SLCO1B1 rs4149056 g.21331549T>C			0.6			0.5			0.3
C/T	13 (100.0%)	0		12 (92.3%)	1 (7.7%)		11 (84.6%)	2 (15.4%)	
T/T	32 (91.4%)	3 (8.6%)		34 (97.1%)	1 (2.9%)		33 (94.3%)	2 (5.7%)	
CYP3A4			>0.9			>0.9			>0.9
*1/*1	39 (92.9%)	3 (7.1%)		40 (95.2%)	2 (4.8%)		38 (90.5%)	4 (9.5%)	
*1/*22	4 (100.0%)	0		4 (100.0%)	0		4 (100.0%)	0	
CYP3A5			>0.9			>0.9			>0.9
*1/*3	2 (100.0%)	0		2 (100.0%)	0		2 (100.0%)	0	
*3/*3	42 (93.3%)	3 (6.7%)		43 (95.6%)	2 (4.4%)		41 (91.1%)	4 (8.9%)	

[1] n (%), [2] Fisher's exact test.

Supplementary Table S4. Incidence of metabolism and nutritional, gastrointestinal and hepatobiliary disorders based on the genotype.

	Metabolism and nutritional disorders			Gastrointestinal disorders			Hepatobiliary disorders		
**Genotype**	**No** N = 45[1]	**Yes** N = 3[1]	***p*-value**[2]	**No** N = 44[1]	**Yes** N = 4[1]	***p*-value**[2]	**No** N = 47[1]	**Yes** N = 1[1]	***p*-value**[2]
ABCB1 rs1045642 g.208920T>C			0.2			0.2			>0.9
C/C	9 (81.8%)	2 (18.2%)		9 (81.8%)	2 (18.2%)		11 (100.0%)	0	
C/T	30 (96.8%)	1 (3.2%)		30 (96.8%)	1 (3.2%)		30 (96.8%)	1 (3.2%)	
T/T	6 (100.0%)	0		5 (83.3%)	1 (16.7%)		6 (100.0%)	0	
ABCB1 rs1128503 g.167964T>C			0.7			0.5			>0.9
C/C	15 (88.2%)	2 (11.8%)		16 (94.1%)	1 (5.9%)		17 (100.0%)	0	
C/T	26 (96.3%)	1 (3.7%)		25 (92.6%)	2 (7.4%)		26 (96.3%)	1 (3.7%)	
T/T	4 (100.0%)	0		3 (75.0%)	1 (25.0%)		4 (100.0%)	0	
ABCB1 rs2032582 g.186947T>G,A			0.7			0.3			>0.9
G/G	17 (89.5%)	2 (10.5%)		17 (89.5%)	2 (10.5%)		19 (100.0%)	0	
G/A + G/T	24 (96.0%)	1 (4.0%)		24 (96.0%)	1 (4.0%)		24 (96.0%)	1 (4.0%)	
A/A	4 (100.0%)	0		3 (75.0%)	1 (25.0%)		4 (100.0%)	0	
ABCG2 rs2231137 g.89061114C>T			>0.9			0.3			>0.9
C/C	42 (95.5%)	2 (4.5%)		41 (93.2%)	3 (6.8%)		43 (97.7%)	1 (2.3%)	
C/T	4 (100.0%)	0		3 (75.0%)	1 (25.0%)		4 (100.0%)	0	
ABCG2 rs2231142 g.89052323G>T			0.2			>0.9			>0.9
G/G	41 (95.3%)	2 (4.7%)		39 (90.7%)	4 (9.3%)		42 (97.7%)	1 (2.3%)	
G/T	3 (75.0%)	1 (25.0%)		4 (100.0%)	0		4 (100.0%)	0	
SLCO1B1 rs4149056 g.21331549T>C			0.2			>0.9			0.3
C/T	11 (84.6%)	2 (15.4%)		12 (92.3%)	1 (7.7%)		12 (92.3%)	1 (7.7%)	
T/T	34 (97.1%)	1 (2.9%)		32 (91.4%)	3 (8.6%)		35 (100.0%)	0	
CYP3A4			>0.9			0.3			>0.9
*1/*1	39 (92.9%)	3 (7.1%)		39 (92.9%)	3 (7.1%)		41 (97.6%)	1 (2.4%)	
*1/*22	4 (100.0%)	0		3 (75.0%)	1 (25.0%)		4 (100.0%)	0	
CYP3A5			>0.9			>0.9			>0.9
*1/*3	2 (100.0%)	0		2 (100.0%)	0		2 (100.0%)	0	
*3/*3	42 (93.3%)	3 (6.7%)		41 (91.1%)	4 (8.9%)		44 (97.8%)	1 (2.2%)	

[1] n (%), [2] Fisher's exact test.

Supplementary Table S5. Incidence of musculoskeletal, vascular and general disorders based on the genotype.

	Musculoskeletal disorders			Vascular disorders			General disorders		
**Genotype**	**No** N = 44[1]	**Yes** N = 4[1]	***p*-value**[2]	**No** N = 29[1]	**Yes** N = 19[1]	***p*-value**[2]	**No** N = 46[1]	**Yes** N = 2[1]	***p*-value**[2]
ABCB1 rs1045642 g.208920T>C			0.7			0.8			0.6
C/C	11 (100.0%)	0		7 (63.6%)	4 (36.4%)		10 (90.9%)	1 (9.1%)	
C/T	27 (87.1%)	4 (12.9%)		19 (61.3%)	12 (38.7%)		30 (96.8%)	1 (3.2%)	
T/T	6 (100.0%)	0		3 (50.0%)	3 (50.0%)		6 (100.0%)	0	
ABCB1 rs1128503 g.167964T>C			>0.9			0.8			>0.9
C/C	16 (94.1%)	1 (5.9%)		11 (64.7%)	6 (35.3%)		16 (94.1%)	1 (5.9%)	
C/T	24 (88.9%)	3 (11.1%)		16 (59.3%)	11 (40.7%)		26 (96.3%)	1 (3.7%)	
T/T	4 (100.0%)	0		2 (50.0%)	2 (50.0%)		4 (100.0%)	0	
ABCB1 rs2032582 g.186947T>G,A			>0.9			0.6			>0.9
G/G	17 (89.5%)	2 (10.5%)		13 (68.4%)	6 (31.6%)		18 (94.7%)	1 (5.3%)	
G/A + G/T	23 (92.0%)	2 (8.0%)		14 (56.0%)	11 (44.0%)		24 (96.0%)	1 (4.0%)	
A/A	4 (100.0%)	0		2 (50.0%)	2 (50.0%)		4 (100.0%)	0	
ABCG2 rs2231137 g.89061114C>T			0.4			>0.9			0.2
C/C	40 (90.9%)	4 (9.1%)		26 (59.1%)	18 (40.9%)		43 (97.7%)	1 (2.3%)	
C/T	3 (75.0%)	1 (25.0%)		3 (75.0%)	1 (25.0%)		3 (75.0%)	1 (25.0%)	
ABCG2 rs2231142 g.89052323G>T			0.3			0.6			>0.9
G/G	40 (93.0%)	3 (7.0%)		25 (58.1%)	18 (41.9%)		41 (95.3%)	2 (4.7%)	
G/T	3 (75.0%)	1 (25.0%)		3 (75.0%)	1 (25.0%)		4 (100.0%)	0	
SLCO1B1 rs4149056 g.21331549T>C			>0.9			0.6			0.5
C/T	12 (92.3%)	1 (7.7%)		7 (53.8%)	6 (46.2%)		12 (92.3%)	1 (7.7%)	
T/T	32 (91.4%)	3 (8.6%)		22 (62.9%)	13 (37.1%)		34 (97.1%)	1 (2.9%)	
CYP3A4			>0.9			0.13			0.2
*1/*1	38 (90.5%)	4 (9.5%)		23 (54.8%)	19 (45.2%)		41 (97.6%)	1 (2.4%)	
*1/*22	4 (100.0%)	0		4 (100.0%)	0		3 (75.0%)	1 (25.0%)	
CYP3A5			>0.9			>0.9			>0.9
*1/*3	2 (100.0%)	0		1 (50.0%)	1 (50.0%)		2 (100.0%)	0	
*3/*3	41 (91.1%)	4 (8.9%)		27 (60.0%)	18 (40.0%)		43 (95.6%)	2 (4.4%)	

[1] n (%), [2] Fisher's exact test.

Supplementary Table S6. Influence of concomitant administration of CYP3A4-inducers on ADRs.

Type of Adverse Reaction	Total Frequency (n=49) N (%)	Concomitant Administration of CYP3A4-Inducers		*p*-Value[1]
		No (n=35)	Yes (n=14)	
Infections and infestations	13 (26.5)	7 (20.0)	6 (42.9)	0.2
Neoplasms	4 (8.2)	2 (5.7)	2 (14.3)	0.6
Nervous system disorders	5 (10.2)	4 (11.4)	1 (7.1)	>0.9
Cardiac disorders	3 (6.1)	2 (5.7)	1 (7.1)	>0.9
Skin and subcutaneous tissue disorders	2 (4.1)	2 (5.7)	0	>0.9
Blood and lymphatic system disorders	4 (8.2)	2 (5.7)	2 (14.3)	0.6
Metabolism and nutritional disorders	3 (6.1)	3 (8.6)	0	0.5
Gastrointestinal disorders	4 (8.2)	3 (8.6)	1 (7.1)	>0.9
Hepatobiliary disorders	1 (2.0)	1 (2.9)	0	>0.9
Musculoskeletal disorders	5 (10.2)	3 (8.6)	2 (14.3)	0.6
Vascular disorders	19 (38.8)	12 (34.2)	7 (50.0)	0.3
General disorders	2 (4.1)	1 (2.9)	1 (7.1)	0.5

[1] Fisher's exact test; Pearson's Chi-squared test.

Supplementary Table S7. Influence of concomitant administration of CYP3A4-inhibitors on ADRs.

Type of Adverse Reaction	Total Frequency (n=49) N (%)	Concomitant Administration of CYP3A4-Inhibitors		*p*-Value[1]
		No (n=32)	Yes (n=17)	
Infections and infestations	13 (26.5)	6 (18.8)	7 (41.2)	0.2
Neoplasms	4 (8.2)	3 (9.4)	1 (5.9)	>0.9
Nervous system disorders	5 (10.2)	3 (9.4)	2 (11.8)	>0.9
Cardiac disorders	3 (6.1)	2 (6.3)	1 (5.9)	>0.9
Skin and subcutaneous tissue disorders	2 (4.1)	2 (6.3)	0	0.5
Blood and lymphatic system disorders	4 (8.2)	2 (6.3)	2 (11.8)	0.6
Metabolism and nutritional disorders	3 (6.1)	2 (6.3)	1 (5.9)	>0.9
Gastrointestinal disorders	4 (8.2)	3 (9.4)	1 (5.9)	>0.9
Hepatobiliary disorders	1 (2.0)	0	1 (5.9)	0.3

Type of Adverse Reaction	Total Frequency (n=49) N (%)	Concomitant Administration of CYP3A4-Inhibitors		*p*-Value[1]
		No (n=32)	Yes (n=17)	
Musculoskeletal disorders	5 (10.2)	2 (6.3)	3 (17.6)	0.3
Vascular disorders	19 (38.8)	14 (43.8)	5 (29.4)	0.3
General disorders	2 (4.1)	2 (6.3)	0	0.5

[1] Fisher's exact test; Pearson's Chi-squared test.
